# Variants in *NGLY1* lead to intellectual disability, myoclonus epilepsy, sensorimotor axonal polyneuropathy and mitochondrial dysfunction

**DOI:** 10.1111/cge.13706

**Published:** 2020-01-30

**Authors:** Daan M. Panneman, Saskia B. Wortmann, Charlotte A. Haaxma, Peter M. van Hasselt, Nicole I. Wolf, Yvonne Hendriks, Benno Küsters, Sjenet van Emst‐de Vries, Els van de Westerlo, Werner J.H. Koopman, Liesbeth Wintjes, Frans van den Brandt, Maaike de Vries, Dirk J. Lefeber, Jan A.M. Smeitink, Richard J. Rodenburg

**Affiliations:** ^1^ Radboud Center for Mitochondrial Medicine, Department of Pediatrics Amalia Children's Hospital Nijmegen the Netherlands; ^2^ Radboud Institute for Molecular Life Sciences, Radboudumc Nijmegen the Netherlands; ^3^ University Children's Hospital Paracelcus Medical University (PMU) Salzburg Austria; ^4^ Institute of Human Genetics Helmholtz Zentrum München Neuherberg Germany; ^5^ Institute of Human Genetics Technische Universität München Munich Germany; ^6^ Department of Neurology, Donders Institute for Brain Cognition and Behaviour Nijmegen the Netherlands; ^7^ Department of Metabolic Diseases, Wilhelmina Children's Hospital Utrecht University Medical Center Utrecht Utrecht the Netherlands; ^8^ Department of Child Neurology, Emma Children's Hospital, Amsterdam UMC ‐ Locatie VUMC and Amsterdam Neuroscience Vrije Universiteit Amsterdam the Netherlands; ^9^ Department of Clinical Genetics Amsterdam UMC ‐ Locatie VUMC Amsterdam the Netherlands; ^10^ Department of Pathology Radboudumc Nijmegen the Netherlands; ^11^ Department of Biochemistry Raboudumc Nijmegen the Netherlands; ^12^ Translational Metabolic Laboratory, Department of Laboratory Medicine Radboudumc Nijmegen the Netherlands

**Keywords:** mitochondrial disorders, NGLY1, OXPHOS enzyme activity, Seahorse respirometry, Whole exome sequencing

## Abstract

*NGLY1* encodes the enzyme N‐glycanase that is involved in the degradation of glycoproteins as part of the endoplasmatic reticulum‐associated degradation pathway. Variants in this gene have been described to cause a multisystem disease characterized by neuromotor impairment, neuropathy, intellectual disability, and dysmorphic features. Here, we describe four patients with pathogenic variants in *NGLY1.* As the clinical features and laboratory results of the patients suggested a multisystem mitochondrial disease, a muscle biopsy had been performed. Biochemical analysis in muscle showed a strongly reduced ATP production rate in all patients, while individual OXPHOS enzyme activities varied from normal to reduced. No causative variants in any mitochondrial disease genes were found using mtDNA analysis and whole exome sequencing. In all four patients, variants in *NGLY1* were identified, including two unreported variants (c.849T>G (p.(Cys283Trp)) and c.1067A>G (p.(Glu356Gly)). Western blot analysis of N‐glycanase in muscle and fibroblasts showed a complete absence of N‐glycanase. One patient showed a decreased basal and maximal oxygen consumption rates in fibroblasts. Mitochondrial morphofunction fibroblast analysis showed patient specific differences when compared to control cell lines. In conclusion, variants in NGLY1 affect mitochondrial energy metabolism which in turn might contribute to the clinical disease course.

## Graphical abstract

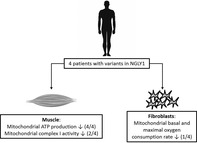

## 1 INTRODUCTION

The clinical spectrum of mitochondrial disorders (MDs) is extremely broad and there are numerous underlying biochemical and genetic defects associated with this type of disorders. While suggestive for a MD, clinical signs like infantile myoclonus epilepsy, intellectual disability, and muscular hypotonia^1^, ^2^) show clinical overlap with other (neuro)muscular and (neuro)metabolic disorders.

This overlap is evident in recently published patients with an impaired function of N‐glycanase 1 (NGLY1). This genetic defect causes a broad clinical spectrum including hypo‐/alacrima, developmental delay, elevated liver enzymes, diminished deep tendon reflexes, and epileptic seizures.^3^, ^4^, ^5^, ^6^ NGLY1 is an enzyme involved in the removal of N‐glycans from glycoproteins, which precedes the proteolytic degradation of glycosylated proteins via the endoplasmatic reticulum‐associated degradation (ERAD) pathway.^7^ In two recent articles, it was suggested that NGLY1 is involved in mitochondrial function in *Caenorhabditis elegans*, mouse, and human and is regulated through nuclear respiratory factor 1 (NRF1).^6^, ^8^

Here we report four patients with biallelic variants in *NGLY1* who presented with myoclonus epilepsy, peripheral neuropathy, and metabolic markers suggestive for mitochondrial dysfunction. Molecular testing revealed further mitochondrial morphological and functional alterations which have not yet been described for this patient group. Taken together, these results provide evidence for a possible role for NGLY1 in mitochondrial function.

## 2 MATERIALS AND METHODS

### 2.1 Cell culture

Fibroblasts were cultured in Medium 199 (M199) (#P04‐07500, Pan Biotech) supplemented with 10% v/v FCS (#10270, Gibco) and 1% v/v penicillin/streptomycin (#15140‐122, Gibco) in a humidified atmosphere with 5% CO_2_ at 37°C.

### 2.2 Pathology and functional mitochondrial measurements in different tissues

Routine histology and histochemistry were performed in muscle following standard methods.^9^ The ATP production from pyruvate oxidation in fresh muscle and the activity of the mitochondrial complexes I to V, citrate synthase, and protein concentration were measured in fresh muscle biopsies, cultured fibroblasts and liver tissue as described previously.^10^

### 2.3 Whole exome sequencing

Whole exome sequencing (WES) and data analysis were performed as described before.^11^, ^12^ In short, exome enrichment was performed using the SureSelect Human All Exon 50 Mb Kit V5 (Agilent). Sequencing was done on a HiSeq4000 (Illumina) with a minimum median coverage of ×80. Read alignment to the human reference genome (GrCH37/hg19) and variant calling was performed at BGI (Copenhagen) using BWA and GATK software, respectively. Variant annotation was performed using a custom designed in‐house annotation. Intronic variants (except for splice sites), synonymous changes, and common variants were filtered and excluded from the initial datasets. Patient data were first analyzed using a custom‐made virtual gene panel containing mitochondrial disease genes (as described in OMIM) as well all other genes known to encode mitochondrial proteins. As no disease‐causing variants were detected, the entire exome was investigated for rare, protein damaging variants. This was done by comparison with the GnomAD dataset, dbSNPv132 and our in‐house variant database with MAF depending on mode of inheritance.

### 2.4 Western blotting

Western Blot analysis was performed on 600*g* supernatant from muscle homogenate and fibroblast homogenates (40 μg per lane) from patients and healthy controls. For fibroblasts homogenates, cell pellets containing 5∙10^6^ cells were resuspended 1% Triton in 10 mM Tris‐HCl (pH 7.6) followed by centrifugation at 4°C 14 000*g*. Protein concentrations in the supernatant were measured using the U/CSF protein kit (Thermo Fischer Scientific) in the Kone‐Lab 20XT semi‐automated platform (Thermo Fischer Scientific, Passau, Germany). Sodium Dodecyl sulfate polyacrylamide gel electrophoresis (SDS‐PAGE) was performed using a 10% Tris‐glycine gel. Western blotting was done on a PVDF membrane and the membrane was probed with rabbit anti NGLY1, 1:400 (PAB23068; Abnova). As a secondary antibody peroxidase conjugated anti‐rabbit, 1:5000 (Genscript, Piscataway) or anti‐mouse, 1:2000 (Dako, Heverlee, Belgium) was used. As a loading control, 1:2000 anti‐SDHA (CII) (ab14715, Abcam) was used for muscle and fibroblasts. The chemiluminescence signal was visualized using the enhanced chemiluminescence kit (ECL, Thermo Fischer Scientific) and the Chemidoc XRS+ system (Biorad, Hercules, California).

### 2.5 Oxygen consumption measurements

Oxygen consumption rates (OCRs) were measured using the Seahorse XFe96 Extracellular Flux analyzer (Agilent). Control and patient primary skin fibroblasts were seeded at 15 000 per well in cell culture medium (M199 supplemented with 10% v/v FCS (Gibco/Life Technologies) and 1% v/v penicillin/streptomycin (Gibco/LifeTechnologies)) and grown overnight at 37°C with 5% CO_2_. One hour before measurement, culture medium was removed and replaced by Agilent Seahorse XF Base Medium complemented with 10 mM glucose (Sigma), 1 mM sodium pyruvate (Gibco), and 200 mM l‐glutamine (Life sciences) and incubated at 37°C, without CO_2_. Basal oxygen consumption was measured four times followed by three measurement cycles after each addition of 1 μM oligomycin A (Sigma), 3, 4, 5, or 6 μM carbonyl cyanide 4‐(trifluoromethoxy) phenylhydrazone FCCP (Sigma), and 0.5 μM rotenone and 0.5 μM antimycin A (Sigma), respectively. One measurement cycle consisted of 3 minutes of incubation and 3 minutes of measuring. OCR was normalized to citrate synthase activity according to the protocol described in Srere et al. (1969) modified for the Seahorse 96 wells format. In short, after completion of OCR measurements, the Seahorse assay medium was replaced by 0.33% Triton X‐100, 10 mM Tris‐HCl (pH 7.6), after which the plates were stored at −80°C. Before measurements, the plates underwent two thaw‐freeze cycles and 3 mM acetyl‐CoA, 1 mM 5,5′‐dithiobis‐2‐nitrobenzoic acid (DTNB), and 10% Triton X‐100 was added. Using a Tecan Spark spectrophotometer (Tecan, Switzerland), background conversion of DTNB was measured at 412 nm and 37°C for 10 minutes at 1 minute intervals. Hereafter, 10 mM of the citrate synthase substrate oxaloacetate was added to start the reaction. Subsequently, the ΔA412 nm was measured again for 10 minutes at 1 minute intervals at 37°C. Citrate synthase activity was calculated from the rate of DTNB conversion in the presence of substrate, from which the background DTNB conversion rate was subtracted, using an extinction coefficient of 0.0136 μmol/cm. Basal respiration was calculated by subtracting the respiration after addition of rotenone and antimycin A from the first four measurements. For each individual cell line, maximal OCR was acquired by titrating FCCP, with a range from 3 to 6 μM. The highest OCR was then used to calculate maximal OCR by subtracting the respiration after addition of rotenone and antimycin A. The average maximal and basal respiration of two control cell lines and individual patient fibroblasts were compared using a Student's *t*‐test; *P* < .05 was considered to be statistically significant. Respirometry are displayed as mean ± SD.

### 2.6 Mitochondrial morphofunctional analysis

Fibroblasts control cell lines included: CT5120 (passage number p24), Control A (p18), and Control B (p21). Patient fibroblast cell lines included: Patient 1 (p = X + 5), Patient 2 (p17), Patient 3 (p19) and Patient 4 (p = X + 14). These cells were cultured in HEPES‐buffered M199 medium (#22340‐087, Invitrogen) supplemented with 10% (v/v) FBS and 1% (v/v) pen/strep in a humidified atmosphere (95% air, 5% CO_2_) at 37°C up to passage number. Cells were grown to 100% confluence before they were seeded at a density of 7000/dish (FluoroDishes; #FD35‐100; World Precision Instruments Ltd., Friedberg Germany). Following 5 days of culturing to allow cell spreading, the M199 medium was replaced by a colorless M199 variant without phenol red (#11043‐023; Invitrogen). Then, DMSO‐dissolved tetramethylrhodamine methyl ester (TMRM; #T668; Life Technologies Thermo Fisher Scientific, Waltham, Massachusetts) was added (15 nM; 25 min) to stain the cells (in the dark; humidified atmosphere; 95% air; 5% CO_2_) at 37°C. Next, dishes were placed on a video‐microscope described in detail previously.^13^ Fluorescence images were acquired in TMRM non‐quenching mode within 15 min after staining^14^ using a ×40 objective, 100 ms exposure time, 540 nm excitation, 560DRLP dichroic mirror and 565ALP emission filter. Extracellular TMRM (15 nM) was present during image recording. Microscopy images were background corrected and processed to allow subsequent calculation of mitochondrial morphofunctional parameters.^15^ In this analysis, only objects larger than 10 pixels were considered to represent mitochondria. Morphofunctional parameters included *Am* (a measure of mitochondrial size in pixels), *AR* (or aspect ratio, a measure of mitochondrial length), *F* (formfactor, a combined measure of mitochondrial length and degree of branching) and *Dm* (density mean, reflecting the mitochondrial TMRM fluorescence intensity). For quality control (*QC*) analysis, a control cell line (CT5120) from a healthy individual was included in all TMRM experiments. If the measured CT5120 parameter values corresponded with those that were previously obtained, the experiment was considered valid and included in the analysis.^15^

### 2.7 Ethics

Written and oral informed consent for diagnostic and research studies was obtained for all subjects in accordance with the Declaration of Helsinki and following the regulations of the local medical ethics committee.

## 3 RESULTS

**Figure 1:**
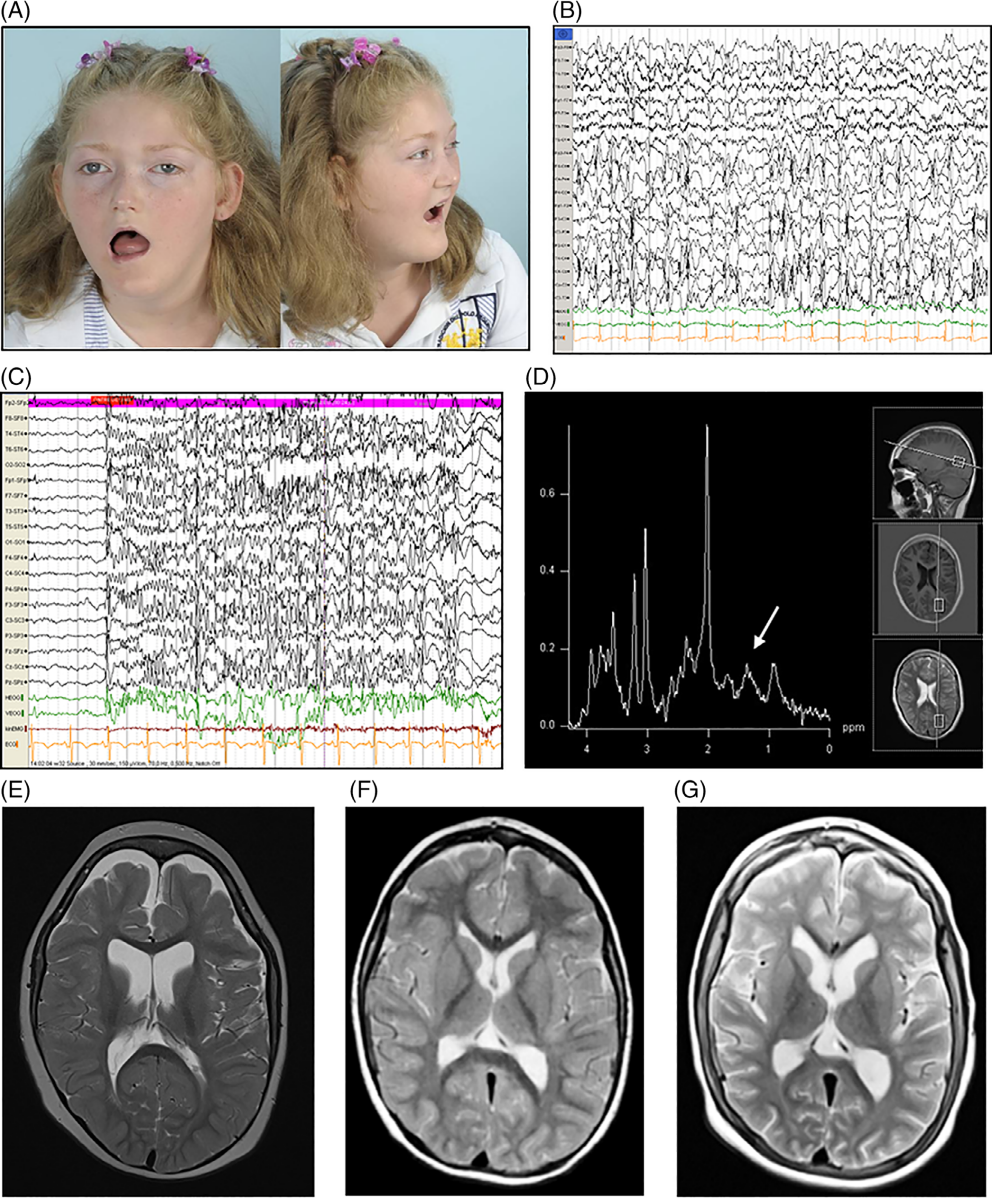
A, Patient 3 at the age of 11 years. Note the mild ptosis, expressionless facies and open mouth (myopathic face), the strabismus, long philtrum, the low implanted large, deformed ears, the midfacial hypoplasia, as well as the pointed chin and short forehead. B, Interictal EEG registration in patient 1 at the age of 7 years showing near‐continuous, multifocal epileptiform discharges with a frontocentral predominance. C, EEG registration in patient 3 at the age of 11 years, demonstrating abundant polyspikes, clinically correlated with frequent, randomly located myoclonic jerks, as well as periodic apneas. D, MR spectroscopy of patient 3 at the age of 11 years, showing mildly elevated lactate (indicated by white arrow) in the occipital white matter. E, Brain MRI, T2 weighted, of patient 1 at the age of 6 years, demonstrating the decreased white matter throughout the brain, and as a result, enlarged CSF compartments. F, Brain MRI, T2 weighted, of patient 4 at the age of 3 years. G, Brain MRI, T2 weighted, of patient 4 at the age of 8 years [Colour figure can be viewed at http://wileyonlinelibrary.com]

Three female patients, two of Dutch and one of Moroccan ancestry, and one male patient of Dutch ancestry were evaluated from an early age onwards because of moderate to severe intellectual disability, developmental delay, muscular hypotonia, and extra pyramidal movements of the extremities (summarized in Table 1). Dysmorphic facial features were observed in patient 3 (Patient 3; Figure 1A). Repetitive metabolic analysis of blood and urine showed mildly elevated serum lactate levels in patients 3 and 4, and elevated serum alanine levels in patient 4. In addition, patient 4 had elevated urinary excretion of Krebs cycle intermediates, patient 1 and 2 had 3‐methylglutaconic aciduria, and patient 3 and 4 lactic aciduria. Elevated liver transaminases were found in patients 1 and 2, while patient 1 also developed liver cirrhosis at a later age. During the course of the disease, three patients developed myoclonic epilepsy that was difficult to treat, encephalopathy, pyramidal tract signs, muscular atrophy of the extremities, and low or absent tendon reflexes. A sensorimotor axonal polyneuropathy was proven by EMG in all four patients. Patient 4 was diagnosed with adrenal insufficiency at age 9 years and has been published.^16^ Patients 1 to 3 showed no signs of adrenal insufficiency to date. Aberrant EEG registrations were observed in patients 1, 3, and 4 (Figure 1B,C). Brain MRI of patient 3 showed no structural abnormalities but MR spectroscopy did show a slightly elevated lactate in the occipital white matter (Figure 1D). During MRS, patient 3 received topiramate to treat epilepsy. Brain MRI showed delayed myelination and a diffuse lack of white matter volume in patient 1 (Figure 1E). Brain MRI of patient 4, performed at age 3 (Figure 1F) and 8 years (Figure 1G), showed incomplete myelination and slowly progressive global atrophy. Patient 1 has deceased at the age of 8 years because of respiratory insufficiency. The family histories of these patients were unremarkable.

**Table 1:** Summarizing previously published patients together with newly identified patients

	Enns et al (*N* = 8)	Caglayan et al (*N* = 2)	Heeley et al (*N* = 1)	Kong et al (*N* = 2)	Patient 1	Patient 2	Patient 3	Patient 4	*N* = 17
Ethnicity	Caucasian	Caucasian			Caucasian	Caucasian	Caucasian	North‐African	
Variants in *NGLY1*	c.1201A>T (p.Arg401*) and c.1201A>T (p.Arg401*) (*N*=5); c.1891del (p.Gln631Serfs*7) and c.1201A>T (p.Arg401*); c.1370dupG (p.Arg458Lysfs*14 and c.1370dupG (p.Arg458Lysfs*14); c.1205_1207del (p.Arg402del) and c.1624C>T (p.Arg542*)	c.1533_1536delTCAA (p.Asn511LysfsX51) and c.1533_1536delTCAA (p.Asn511LysfsX51)	c.347C>G (p.S116*) and c.881+5G>T (p.?)	402_403del (c.1205_1207del) and c.1570C>T (p.R524X); c.1604G>A (p.W535X) and c.1910delT (p.L637X)	c.1201A>T (p.Arg401*) and c.849T>G (p.Cys283Trp)	c.1201A>T (p.Arg401*) and c.1201A>T (p.Arg401*)	c.1201A>T (p.Arg401*) and c.1067A>G (p.Glu356Gly)	c.1837del (p.Gln613fs) and c.1837del (p.Gln613fs)	
ID/DD	8/8	2/2	+	2/2	+	+	+	+	17/17
Involuntary movements	8/8	2/2	+	2/2	+	−	+	+	16/17
Muscular hypotonia	8/8	1/2	+	ND	+	+	+	+	14/17
Myoclonus epilepsy	ND	ND	+?	0/2	+	−	+	+	3/6
Seizures	4/8	1/2	+	0/2	+	−	+	+	9/17
EEG abnormalities	7/8	1/2	+	ND	+	−	+	+	12/17
Polyneuropathy	3/3	2/2	+	0/2	+	+	+	+	10/12
Dysmorphic features	4/8	1/2	+	0/2	+	+	+	?	9/17
Small hands and feet	4/8	0/2	−	0/2	+	+	+	−	7/17
Alacrima/hypoalacrima	7/8	2/2	+	0/2	−	+	−	−	11/17
Corneal ulcerations/scarring	4/8	2/2	+	0/2	+	−	−	−	8/17
Strabismus	4/8	1/2	+	0/2	+	+	+	−	9/17
Elevated liver transaminases	6/7	1/2	+	2/2	+	+	−	−	12/16
Liver fibrosis	2/6	0/2	+	0/2	+	−	−	−	4/15
Elevated lactate	4/6	0/2	−	1/2	+	−	+ (serum, CSF)	−	7/15
Elevated serum alanine	ND	ND	ND	ND	−	−	−	+	1/4
Abnormal urinary organic acids results	ND	ND	ND	ND	+ (3‐methylglutaconic aciduria)	+ (3‐methylglutaconic aciduria)	+ (lactate)	+ (lactate, Krebs cycle intermediates)	4/4
Mitochondrial disease criteria^17^					5/12 (probable MD)	6/12 (probable MD)	6/8^a^ (probable MD)	6/8^a^ (probable MD)	N/A

Abbreviations: DD, developmental delay; ID, intellectual disability; NA, not applicable; ND, not determined.

a No data on histology.

**Figure 2:**
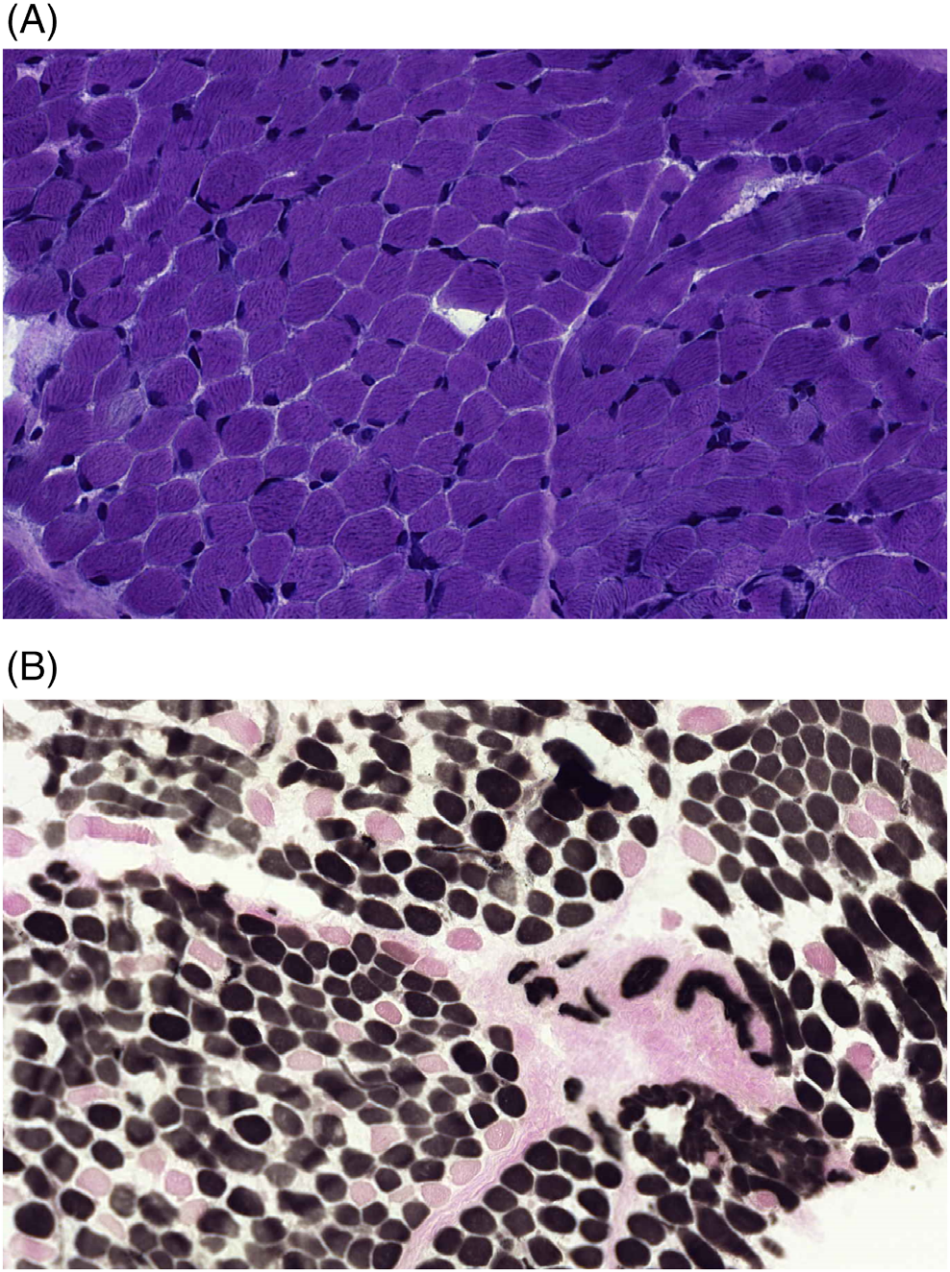
A, Haematoxylin‐phloxin staining of a muscle biopsy of patient 1, showing in general a normal morphology, but sporadically some smaller fibers. There are no basophilic fibers. B, ATPase staining (pH 4.2), showing a predominance of type 1 fibers (85% of all fibers; darkly stained fibers) [Colour figure can be viewed at http://wileyonlinelibrary.com]

As the clinical and metabolic symptoms were suggestive for a mitochondrial disease, all four patients underwent a muscle and skin biopsy in order to investigate mitochondrial function. All patients were clinically stable and none of them received any additional nutritional supplements such as vitamins at the time of muscle biopsy and during the measurements of metabolic markers and MRI. Patient 3 received topiramate at the time of the muscle biopsy. The biochemical evaluation revealed a clearly reduced mitochondrial ATP production from the oxidation of pyruvate and malate, that was around 50% of the lowest reference value (or 25% of the mean reference value) in all four patients (Table 2), a clear sign of a mitochondrial defect in all patients investigated. Additionally, patients 1 and 3, showed a moderately reduced activity of the respiratory chain enzymes I and III, and enzymes I, II, and II + III, respectively. Evaluation of the OXPHOS enzyme activity in cultured fibroblasts was normal in all patients. Histological analysis of all muscle biopsies showed no abnormalities in patients 2 and 3. Patient 1 showed a dominance of type I fibers and excessive glycogen storage (Figure 2A,B). The latter was also present in patient 4.

**Table 2:** Evaluation of the oxidative phosphorylation system in fresh muscle and cultured fibroblasts

	Parameter	P1	P2	P3	P4	Unit
Muscle	ATP + CrP production rate	**17.1** (42.1‐81.2)	**8.3** (15.4‐30.2)	**21.5** (42.1–81.2)	**8.6** (15.4–30.2)	nmol ATP/h.mU CS
	Complex I	**50** (70‐251)	73 (47‐154)	**56** (70‐251)	55 (47‐154)	mU/U CS
	Complex II	360 (335‐749)	206 (134‐354)	**268** (335‐749)	176 (134‐354)	mU/U CS
	Complex III	**2008** (2200‐6610)	1068 (696‐1756)	2430 (2200‐6610)	954 (696‐1756)	mU/U CS
	Complex IV	1210 (810‐3120)	785 (470‐1842)	1440 (810‐3120)	889 (470‐1842)	mU/U CS
	Complex V	205 (169‐482)	422 (161‐711)	236 (169‐482)	409 (161‐711)	mU/U COX
	Complex II + III	392 (300‐970)	232 (176‐492)	**246** (300‐970)	209 (176‐492)	mU/U CS
	Citrate synthase	52 (37‐162)	**65** (84‐365)	121 (37‐162)	146 (84‐365)	mU/mg protein
Fibroblasts	Complex I	122 (100‐310)	289 (163‐599)	221 (100‐310)	275 (163‐599)	mU/U CS
	Complex II	728 (520‐845)	423 (335‐888)	784 (520‐845)	747 (335‐888)	mU/U CS
	Complex III	1446 (1320‐2610)	915 (570‐1383)	1690 (1320‐2610)	724 (570‐1383)	mU/U CS
	Complex IV	692 (680‐1190)	549 (288‐954)	842 (680‐1190)	431 (288‐954)	mU/U CS
	Complex V	ND	711 (193‐819)	578 (232‐1439)	666 (193‐819)	mU/U CS
	Complex II + III	279 (110‐470)	282 (128‐534)	206 (110‐470)	287 (128‐534)	mU/U CS
	Citrate synthase	228 (144‐257)	364 (151‐449)	238 (144‐257)	312 (151‐449)	mU/mg protein

*Note*: Abnormal results in bold, reference values are between brackets.

These combined observations suggested a mitochondriopathy and subsequently, molecular genetic analysis was performed, including mtDNA sequencing and WES. No variants were identified in any of the known mitochondrial disease genes. Instead, in all four patients, rare biallelic variants in *NGLY1* (NM_018297.3) were detected. In patient 2, the previously reported disease causing variant c.1201A>T (p.(Arg401*)) was found in homozygous state. This variant was also found in heterozygous state in patients 1 and 3. Additionally, both patients carried a missense variant: in patient 1; c.849T>G (p.(Cys283Trp)), and in patient 3; c.1067A>G (p.(Glu356Gly)). In patient 4, the homozygous variant c.1837del was detected, introducing a frameshift and premature stop codon (p.(Ile613Phefs*6)) in the last exon of NGLY1.^16^ The respective parents were all carriers of one of the variants, proving that both alleles carried variants in all four patients. All missense variants are predicted to be pathogenic by SIFT, Polyphen and MutationTaster.^18^, ^19^, ^20^ These results lead to reverse phenotyping of our patients. Only one of our patients (patient 1) had corneal ulcerations, hypolacrima was observed in patient 2. Small hands and feet were noticed in patients 1 and 3.

**Figure 3:**
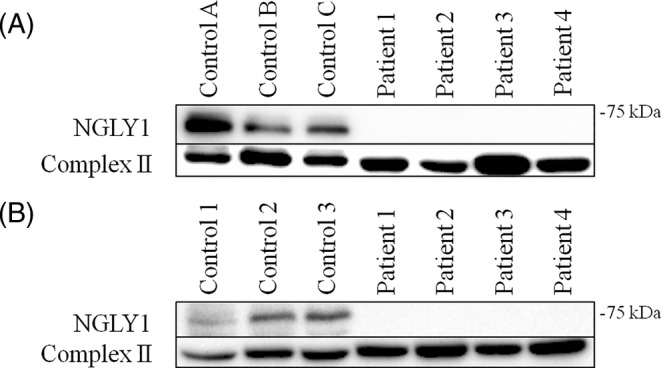
Western blot analysis. A, Immunoblot analysis of NGLY1 in muscle extracts from four patients and three controls. The anti‐NGLY1 antibody stains a 74‐kDa band in the three controls, whereas this band is absent in the four patients. Anti‐SDHA (complex II) was used as loading control. B, Immunoblot analysis of NGLY1 in fibroblasts from four patients and three controls. Similar to the muscle sample, a NGLY1 band is present in the control samples, whereas this band is absent in the four tested patients. Anti‐SDHA (complex II) was used as loading control

**Figure 4:**
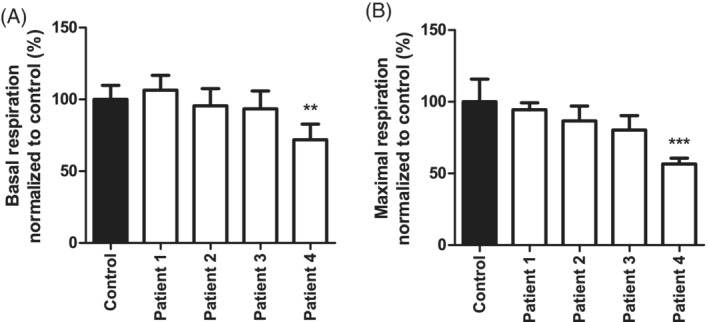
Oxygen consumption measurements. Patient 4 shows a reduced basal and maximal cellular respiration showed a reduction in both basal respiration (*P* < .01) and maximal respiration (*P* < .001) in patient 4 only (n = 3). Oxygen consumption rates were normalized to citrate synthase activity

Because of the observed mitochondrial biochemical defects in all patients, we investigated whether this could be connected to the NGLY1 protein levels. Western blot analysis showed that the NGLY1 protein was absent in all four patients in muscle tissue as well as in cultured skin fibroblasts (Figure 3). OCRs are commonly used as a parameter for mitochondrial function. Here, Seahorse respirometry was used to assess mitochondrial respiration in control and patient fibroblasts. A significant decrease in both basal respiration and maximal respiration was observed in patient 4 (Figure 4A,B). No significant differences were found in the other patients.

**Figure 5:**
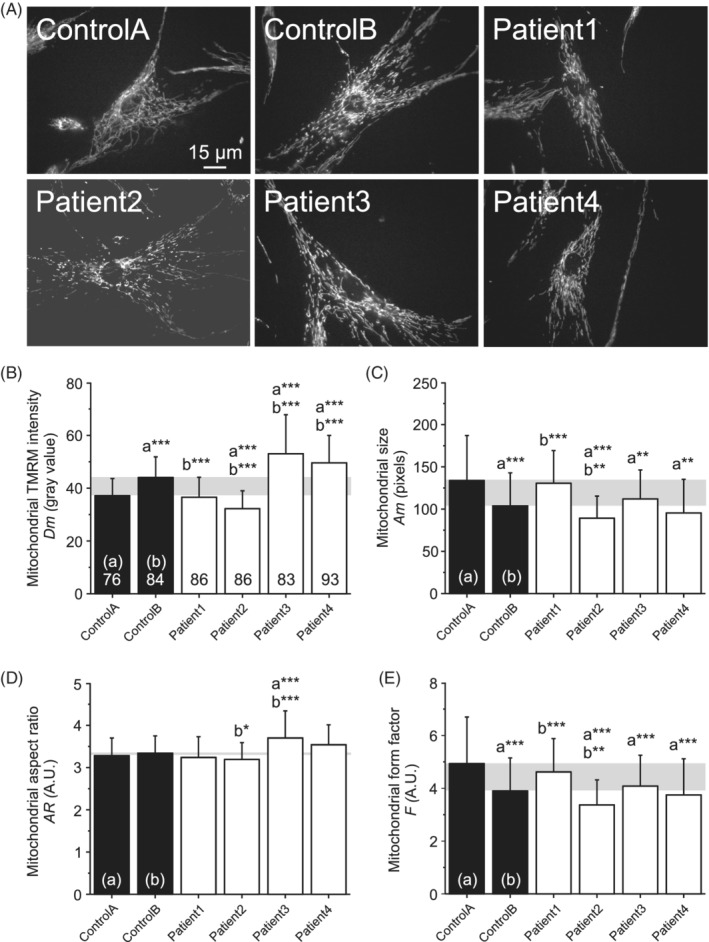
Mitochondrial membrane potential and morphology. A, Typical examples of TMRM‐stained fibroblasts from control individuals (Control A, Control B) and patients (Patient 1, Patient 2, Patient 3, Patient 4). B, Average mitochondrial TMRM fluorescence signal (Dm) in the fibroblasts cell lines. The gray box reflects the difference between the two control cell lines (Control A, Control B). C, Same as panel B but now for the average mitochondrial size (Am). D, Same as panel B but now for the average mitochondrial aspect ratio (AR). E, Same as panel B but now for the average mitochondrial form factor (F). Statistics: Images were contrast‐optimized for visualization purposes. Quantification was performed on the original images. Mean values + SD were calculated for each condition for the number of cells indicated in panel B, measured in three independent experiments. An independent Student's *t*‐test was used for statistical analysis (*P < .05; **P < .01; ***P < .001). The total number of mitochondrial objects analyzed for each condition equaled: 7145 (Control A), 114 212 (Control B), 8859 (Patient 1), 10 645 (Patient 2), 8744 (Patient 3) and 12 101 (Patient 4)

Changes in mitochondrial function and morphology often occur in parallel.^21^ To determine whether mitochondrial membrane potential and morphology were affected in the patient cells we applied morphofunctional analysis.^15^ In this approach, cells are stained with the fluorescent cation TMRM that accumulates in the mitochondrial matrix in a membrane potential (Δ*ψ*)‐dependent manner (Figure 5A). Following live‐cell microscopy, images of TMRM‐stained cells are processed to allow subsequent extraction of mitochondrial morphofunctional parameters. Here we quantified *Dm* (reflecting the mitochondrial TMRM fluorescence intensity; a measure of Δ*ψ*), *Am* (a measure of mitochondrial size), *AR* (aspect ratio; a measure of mitochondrial length), *F* (formfactor; a combined measure of mitochondrial length and degree of branching). In this analysis, the patient cell lines were compared with two control cells from donors of similar age (both 5 years of age). This analysis revealed that *Dm* was lower than control in cells from patient 1 and patient 2 (Figure 5B), suggesting a less negative (partially depolarized) Δ*ψ*. In contrast, *Dm* was higher than controls in patient 3 and patient 4 (Figure 5B), suggesting a more negative (hyperpolarized) Δ*ψ*. Patient 1 and patient 3 displayed a similar *Am* value relative to control, whereas this parameter was reduced in patient 2 and patient 4 (Figure 5C). This suggests that mitochondrial size is normal in patient 1 and 3 but reduced in patient 2 and 4. In general, the patient cells displayed no major alterations in *AR*, although this parameter was increased in patient 3 (Figure 5D). Finally, *F* appeared reduced in patient 2, patient 3 and patient 4, but was normal in patient 1 (Figure 5E). The latter result (combined with the results of Am and AR) suggests that mitochondria are smaller and less branched in patient 2 and patient 4, whereas this is not the case for patient 1 and patient 3.

## 4 DISCUSSION

WES is widely and successfully used to identify the causative genetic defects in patients with all kinds of clinical signs and symptoms. This often leads to a broadening of the clinical spectrum of known disorders. There is a growing number of disorders in which mitochondrial dysfunction is observed but the primary defect cannot be directly linked to known mitochondrial proteins or processes.^22^ The four patients described in this study presented with a multi‐system disorder including sensorimotor axonal peripheral neuropathy, myoclonic epilepsy which were highly suggestive for a mitochondrial disorder, even more so when taking the metabolic alterations in blood and urine into account (Table 1). Previous studies indicate that some of the NGLY1 patients were initially suspected to have a mitochondrial disorder. However, no biochemical evidence for this was found.^3^, ^4^, ^5^, ^6^, ^8^ Additionally, a recent study assessing musculoskeletal manifestations and management thereof in 29 patients with a NGLY1 deficiency was published.^23^ However, it is not clear which patients have been published before and since genotypes were not included, these patients were not summarized in Table 1. In this study, we show that all patients have clear signs of mitochondrial dysfunction at a biochemical level in muscle (Table 2).

Western blot analysis showed an absence of NGLY1 protein expression in patient fibroblast as well as muscle biopsies, indicating that the *NGLY1* genetic variants in these patients result in protein instability or degradation and are therefore likely disease causing. NGLY1 has been characterized as an enzyme involved in the ERAD pathway but the enzyme has been implicated to be involved in mitochondrial function.^6^, ^7^, ^8^ To further dissect the molecular mechanism by which NGLY1 influences mitochondrial energy metabolism we assessed oxygen consumption and mitochondrial morphofunction as a readout for mitochondrial functioning. Patient 4 showed a significant decrease in both basal and maximal respiration indicating mitochondrial dysfunction in this specific patient fibroblast cell line suggesting mitochondrial dysfunction. Conversely, patients 1, 2, and 3 showed no significant decrease in basal and maximal OCRs. Our measurements contradict the previous findings in Kong et al, where researchers found that this particular variant did cause a reduction in basal and maximal respiration.^6^

Mitochondrial morphofunction analysis showed various differences between control and patient fibroblasts. For instance, TMRM intensity, a marker for mitochondrial inner membrane potential, was shown to be increased in patients 3 and 4, indicating hyperpolarisation, but decreased in patients 1 and 2, indicating hypopolarisation. No consistent difference in any parameter could be observed when comparing control and patient fibroblasts like suggested in a previous publication.^8^ Moreover, no distinct morphofunctional phenotype, differentiating patient 4 from the other patients was observed that could possibly explain the reduced maximal respiration. Although the molecular characterization showed variable differences in mitochondrial morphofunction and a decrease in basal and maximal respiration in one patient, no reduced OXPHOS enzyme activities were observed in any of the patient fibroblasts. Although the molecular characterization showed variable differences in mitochondrial morphofunction and a decrease in basal and maximal respiration in one patient, no reduced OXPHOS enzyme activity were observed in any of the patient fibroblasts. It is unlikely that the mitochondrial abnormalities predominantly seen in muscle are secondary to for example, malnutrition^24^ or disuse,^25^ as there was no indication of malnutrition of the patients in the current cohort, nor did the muscle histology show signs of disuse.

To date, the proteins that make up the OXPHOS system have never found to be N‐glycosylated. This might suggest that NGLY1 influences mitochondrial function in a secondary manner.^26^ It has been shown that AMFR, an E3‐ubiquitin ligase involved in the ERAD pathway, localizes to mitochondria associated ER membranes and plays a role in the degradation of Mfn1 and Mfn2 mitochondrial fusion proteins.^27^ This might indicate that NGLY1 is involved in the degradation of mitochondrial outer membrane proteins. Furthermore, it was shown that mitochondrial function is influenced in NGLY1 patients via glycosylated Nrf1.^28^ This article suggests that the mitochondrial network, visualized with an anti‐body directed against Hsp60, is fragmented in patient fibroblasts and *Ngly1* −/− mouse embryonic fibroblasts. The researchers suggest that the mitochondrial network fragmentation is due to absence of NGLY1 which alters the glycosylation of Nrf1, hampering its function and resulting in a fragmented mitochondrial network.^28^ However, we did not note fragmentation of the mitochondrial network when assessing the mitochondrial morphology in patient fibroblasts as observed previously.^8^

To date, 13 patients have been investigated with a pathogenic variant in NGLY1 and with clinical signs and symptoms suggestive of a mitochondrial disorder, including intellectual disability, involuntary movements, and muscular hypotonia.^3^, ^4^, ^5^, ^6^ However, no conclusive biochemical evidence for a mitochondrial defect has been provided yet. Here, we describe four patients that show isolated or combined defects in the OXPHOS enzymes combined with a reduction in ATP production in muscle, strongly suggestive of a mitochondrial disorder. Differences were observed in mitochondrial function in patient fibroblasts, showed that there is a functional relationship between NGLY1 and mitochondria. However, further investigation is needed to further dissect the molecular mechanism connecting NGLY1 to mitochondrial function.

## CONFLICT OF INTEREST

J.S. is, next to this Radboudumc position, founding CEO of Khondrion. The company was not involved in this manuscript. W.J.H.K. is a scientific advisor of Khondrion BV (Nijmegen, the Netherlands) and of Fortify Therapeutics (Palo Alto, USA). These SMEs had no involvement in the data collection, analysis and interpretation, writing of the manuscript, and in the decision to submit the manuscript for publication.

## Data Availability

The data that support the findings of this study are available upon request from the corresponding author.
